# Undergraduate Public Health Capstone Course: Teaching Evidence-Based Public Health

**DOI:** 10.3389/fpubh.2016.00070

**Published:** 2016-04-18

**Authors:** Veronica Eileen Fitzpatrick, Christen Mayer, Barry R. Sherman

**Affiliations:** ^1^University at Albany School of Public Health, State University of New York, Albany, NY, USA

**Keywords:** undergraduate public health education, evidence-based public health, undergraduate instruction, bachelor of science public health

## Abstract

The University at Albany School of Public Health has offered a Bachelor of Science in Public Health (BSPH) degree for the past 7 years. The final requirement of the BSPH degree is a capstone evidence-based public health class designed to culminate the degree program. This capstone course is framed by identifying a public health problem and creating a literature review based on this problem. The issues are selected through collaboration between the students and instructors. Developmental and analytical tools necessary to complete the literature review are provided throughout the semester. By the end of the course, students achieve the necessary competencies and skills to identify a public health problem, analyze information from peer-reviewed literature, and synthesize the relationship between a health issue and its correlated outcome. Successes were measured through achievement of core BSPH competencies, quality of final paper and presentation, and qualitative data gleaned from end of semester self-reported student surveys.

## Introduction

Over the last decade, public health education has witnessed the birth and proliferation of undergraduate public health degrees. The University at Albany (UA) School of Public Health’s Bachelor of Science in Public Health (BSPH) was launched in 2007. This program was designed to serve as a gateway to graduate public health education and culminates in a capstone course. Existing literature describes demand for undergraduate bachelor degrees in public health, although there remains uncertainty in what students can do with this degree (1). There is also uncertainty in what role an entry level public health professional can serve in the public health workforce (2, 3). As such, it was imperative the capstone experience at UA prioritize developing analytical thinking and evidence-based writing skills. How these skills were conveyed and practiced has evolved with each capstone offering. This article outlines the process for successful fulfillment of undergraduate public health learning outcomes and recommended critical elements of an undergraduate major in public health. The BSPH program at the UA has evolved to include elements articulated in the Association of Schools and Programs of Public Health (ASPPH): *Framing the Future: Second Hundred Years of Education in Public Health* (4).

Within the ASPPH Undergraduate Public Health Learning Outcomes Model (2012), the capstone writing course is directly responsive to Domain 2: Intellectual and Practical Skills and Domain 4: Integrative and Applied Learning. The particular skill areas in focus are communications (oral and written) and information literacy. A capstone evidence-based public health course that satisfies the accreditation criterion for a culminating experience to integrate, apply, and synthesize knowledge through cumulative and experiential activities is described here. The final product is a thoroughly researched literature review to confront a public health problem.

## Background

The University at Albany BSPH degree program has four concentrations: policy and management, epidemiology, social behavior and community health, and biomedical and environmental. The degree sequence includes the objectives of nurturing critical thinking, analysis, and synthesis of information, while recognizing the historical and societal context of current trends in public health.

Students complete foundational classes before choosing a concentration. BSPH majors must successfully complete the following:

- Introduction to Public Health,
- Concepts in Epidemiology,
- Global Environmental Issues and their Effect on Human Health,
- Epidemiology and Biostatistics,
- Promoting Healthy People and Communities,
- How U.S. Health-Care Works: myths and realities, and
- Capstone: evidence-based public health.

The course described here is “*Capstone: Evidence-Based Public Health*.” This course is the culminating experience of the undergraduate program. The course teaches the graduating BSPH candidate how to use evidence-based decision-making to address a public health problem by providing practical guidance on how to find and assess the quality of available evidence and write a comprehensive literature review. These objectives are achieved through lectures, practical exercises, group learning, and class presentations. Learning how to analyze information from peer-reviewed literature is the main learning objective of this class.

## Curriculum and Course Design

At course completion, students will have necessary competencies and skills to (1) identify public health problems, (2) analyze information from peer-reviewed literature, and (3) synthesize the relationship between an issue and its correlated outcome. The duration of each section has been largely variable and ultimately left to the discretion of the instructor. However, each section up to “measurement” (below) has been completed by midway through the semester, leaving the bulk of the writing to the second half. Every semester the class has followed a similar timeline:

- Introduction to the concept of exposure and outcome relationship,
- Topic assignments,
- Exposure and outcome articles for each topic,
- Library day,
- What is literature?
- Measurement,
- Cultural competence/ethics in research/engaging a community,
- Target population/stakeholders,
- Critical analysis and prevalence data,
- Review of statistics and study design, and
- Data in the media.

The course begins by introducing students to the concept of evidence-based public health.

The BSPH program relies heavily on classroom-based knowledge, so the capstone is specifically designed to expose students to an evidence-based methodology. Typical class enrollment is 20–25 students. The course is offered annually; accommodating additional students would work best by offering additional sections.

Evidence-based public health is the development, implementation, and evaluation of a public health topic intended to lead to effective interventions, programs, and policies (5). Through application of scientific principles, including systematic uses of data, appropriate employment of social and behavioral science theories, and program planning models that students begin to understand the value of evidence-based research (5). Students must first understand the concept of evidence-based research before the wide range of public health topics are introduced to allow students to identify and explore public health problems.

### Identify Public Health Problems

Instructors have used various methods to select public health issues that would stimulate interest and enthusiasm. These methods have included (1) students choose their issue, (2) issues are assigned, and (3) “umbrella topics” are assigned. For example, under the umbrella of “maternal and child health” a student might choose “mother to child transmission of HIV.” See Table 1, for example.

**Table 1:** **Example of student exposures and outcomes**.

Exposure	Outcome
Maternal stress	Pre-term birth
Air pollution	Low birth weight
Gender discrimination	Depression
Salmonella	Development of Reiter’s syndrome
Low socioeconomic status (SES)	Increase in food borne illnesses
Depression	Cardiovascular disease

Each method has successes and challenges. Letting students pick topics generates excitement; however, students who pick an association with which they are already familiar tend to assume a relationship between the issue and its correlated outcome that may not be evidence based. Student assessments showed allowing students to pick topics was preferred. A large component of the class involved teaching students to use literature to drive their decision-making processes, which becomes a challenge when students assume a relationship. Assigning student issues eliminates assumptions about relationships between the issue and its correlated outcome at the cost of potentially giving students topics in which they do not have interest. The class teaches skills that can be applied to any public health topic, so assigning a topic is more problematic in the beginning of the class. Furthermore, by either assigning a specific topic or letting a student choose a topic under a larger umbrella of topics exposes students to public health issues that they may not have previously encountered. Both methods have resulted in similar final products, however, assigning topics to students requires more work for the instructor and, as stated above, is the less preferred method of students.

The class uses the epidemiological concept of exposure and outcome to simplify the relationship between a particular health problem and its associated outcome. The term risk factor is often used to describe an exposure variable, although this is not always accurate and often comes across as limiting. The term outcome is very broad and for the purposes of the class is defined as disease, state of health, health-related event, or death. In some studies, there may be multiple outcomes, although for the purposes of this course, students are limited to one outcome to simplify the concept.

In the course’s original design, a primary objective of the first few classes was to develop a concise issue statement from the selected topic, after solidifying the concept of evidence-based public health. However, in later years, this timeline was changed, as it became apparent that students were developing an issue without utilizing the literature to define the issue. It was determined that demonstrating to students how to critically read and analyze peer-reviewed journals from the outset was the most effective way for them to comprehend evidence-based public health. This section demonstrated that students were able to develop intellectual skills of inquiry and analysis, and critical and creative thinking.

### Analyze Information from Peer-Reviewed Literature

Learning how to analyze information from peer-reviewed literature is the objective of this course. The final assignment requires demonstration of competence in synthesis and analysis of empirical information in the literature review and oral presentation. To introduce this topic, the instructor guides the class through easy to understand journal articles as a collective activity. The class is tasked with reading two journal articles and questioned about the exposure, outcome, research question, methodology, and conclusion.

Guiding students through a literature search is also an essential component of the course. Each year, the class dedicates one full session to learning the university library system. The university’s science librarian presents on searching for journal articles, requesting articles, and general services. Students have responded very favorably to this every year it had been included. Student evaluations state that demonstrating a tangible way of finding primary literature was a highlight of the class.

Several class sessions are designed to ensure that the students will have the quantitative and information literacy skills necessary to understand the data presented in the articles that they have found and incorporate these data into their assignments in a meaningful way. The intention of these sessions is to teach the students to apply mathematical and comprehension skills to problem solving. For example, students are instructed to compare data presented in the tables of each article to the author’s written conclusion. If there are discrepancies between the data and the conclusions, then students are instructed to pragmatically evaluate the information presented in each the article.

To examine whether the students were able to understand the basics of a journal article, a take home exam was administered. The exam consisted of students finding one journal article and answering questions about that article. The article does not have to be about the students’ previously determined topic, although journal quality is mentioned as part of the introduction to literature section, the only requirements for the exam is that the article contain original research and is peer-reviewed journals. The questions for the exam are as follows:

1. What is the author’s primary research question?
2. What is the exposure and how is it measured?
3. What is the outcome and how is it measured?
4. What is the author’s conclusion?
5. What are the limitations within the study?

The results of this exam offer instructors an indication of how well students are grasping the concept of peer-reviewed literature and how able they are to dissect various components of journal articles. Teaching the fundamentals of the literature reviews is essential to a successful final project. Once the instructor feels comfortable with the class’s ability to understand the literature, he or she can confidently move forward with the course content. This section demonstrated that students were able to develop intellectual and practical skills of inquiry and analysis, critical and creative thinking, quantitative literacy, information literacy, and problem solving.

### Synthesize the Relationship between a Health Issue and Its Correlated Outcome

As stated previously, the capstone assignment of this class is a literature review. It is imperative that students understand the difference between a literature review and a research paper. Most undergraduates are accustomed to research papers where they simply report what they read. By contrast, the literature review involves critical thinking about methods and comparative analysis among various perspectives on the same topic. The final written product should be in the student’s own voice, backed by evidence, not simply a collection of published author conclusions. The purpose of the final written product is to offer an overview of significant literature published on a topic while addressing any gaps in current research. Instructors guide students regarding the organization of the paper by breaking down each major component.

One suggested organizational framework is:

- Background information on the topic, population, and region;
- Exposure background;
- Outcome background;
- Evidence of the association between the exposure/outcome; and
- Limitations.

Students are required to submit a spreadsheet of at least 10 articles related to their topics, organized by journal, authors, research question, exposure, outcome, conclusion, and limitations. This requirement ensures that the students are collecting appropriate articles in a timely manner. Next, a first draft is due and thoroughly edited to guide students toward a high quality final product. In the early years of the course, a draft was not required and lacking this element negatively impacted the quality of the final product. Proper citation is also addressed. Finally, the literature review is submitted and graded for content, grammar, and fulfillment of the assignment. In all student evaluations, it was reported that being taught to write a literature review is the most successful component of the class and develops a tangible skill. This section showed that students were able to demonstrate application of knowledge, skills, and responsibilities to new settings and complex problems.

## Student Assessment

Overall assessment of student learning and analysis was achieved by the following.

Dissection of Existing Peer-Reviewed Literature

Assignments are given in which students identify a research question, exposures and outcomes under study, target population, inclusion and exclusion criteria, authors’ conclusions, and described limitations.

Critical Analysis of Literature Themes Spreadsheet

Segmented approach to clearly illustrate research themes and conclusion patterns among a variety of studies on a related topic from multiple publications.

Literature Review

The comprehensive literature review is assigned and submitted in stages: outline with headings, introduction, full review with proper citations, and an edited revision. The final literature review is expected to contain: background, explanation of exposure, explanation of outcome, description of the association between the exposure and outcome, identification of at risk population, summary of literature themes, conclusions, limitations, and public health significance.

Oral Presentation of Literature Review

Emphasizes the importance of the ability to effectively communicate and disseminate scientific findings to a broad audience.

## Lessons Learned

Presentation of learning objectives in a tangible way has resulted in student understanding of secondary research and development of literature review writing skills. Successes are measured through achievement of core BSPH competencies and qualitative data gleaned from end of semester self-reported student surveys. Mastery of the core competencies is reflected through the quality of comprehensive literature reviews presented at the completion of the course. Progress was observable as students advanced through the stages of the literature review demonstrated by improved communication of concepts and comprehension of evidence-based topics and associations.

Student success was evident to the instructors in the quality of the products created and in the majority of student attitudes and level of course satisfaction. Early in each semester, students communicated frustration and doubt regarding the learning goals. However, understanding of the material was demonstrated through increasingly high quality performance within semesters, as each competency and skill was learned. This success was most evident in the transformation of the draft literature reviews into the final product. The drafts consistently showed student challenges and opportunities for improvement. With individualized instructor feedback and completion of all the learning exercises, the final products showed comprehension and skill development. At the completion of the course, many students verbalized a fully realized sense of accomplishment and the acquisition of a new ability.

Student surveys from 7 years of course delivery showed the following self-reported learning and skill development:

- Scientific writing ability,
- Familiarity with data presentation,
- Understanding of research tools,
- Clarification of exposure/outcome relationships,
- Differentiation between issue associations and singular topic descriptions,
- Knowledge of independent/dependent variable, and
- Identification of appropriate peer-reviewed sources.

Developing a curriculum to encompass developing all the tangible skills necessary to succeed in the public health workforce would be ideal. An effort to that effect was made in the early iterations of the course by including program planning and evaluation assignments. These assignments did not produce quality products from the students and did not consistently align with the evidence-based theme of the course. Student programmatic designs consistently included unfounded assumptions and anecdotal information, which are antithetical to the learning goals of the course. The results of the planning and evaluation assignments demonstrated the need to focus strictly on incorporating relevant evidence in public health decision-making and planning. A full semester is necessary to successfully achieve the described learning objectives of identifying and synthesizing peer-reviewed evidence, outlining and writing the literature review, and presenting a final product. Evidence-based program planning and evaluation should be considered for a corequisite course in the designs of BSPH curriculums.

There were barriers to learning that was identified throughout the evolution of the course. As an example, non-native English speaking students demonstrated challenges in a class that relies heavily on scientific reading and writing in English. Arrangements were made for these students to visit the UA Writing Center in order alleviate any stresses associated with language barriers. Additional office hours were also made available in these instances. In addition, time management and an ability to synthesize large quantities of information presented barriers throughout the years the class has been available. These barriers were addressed by holding the class in a computer lab to allow for literature searches and writing to take place under the direct supervision of the instructors and with the development of Excel spreadsheet categorization tool.

## Conclusion

The UA BSPH degree program is designed to serve as a gateway to graduate public health education and public health careers; therefore, it was evident that an essential component of the course would be to develop skills through analytical thinking and evidence-based writing. Seven years of completed literature reviews and end of semester surveys have shown success is achieved through student empowerment and a stepwise approach to secondary data analysis. Students were able to achieve two ASPHH domains by the end of the class: (2) Intellectual and Practical Skills and (4) Integrative and Applied Learning, which are key components to successful public health graduate studies and careers. Additionally, development of research, writing, and oral presentation skills allowed students graduating with the BSPH degree to contribute to the public health field in relevant and meaningful ways.

Given the limited time of an undergraduate semester, the resources of the class are concentrated on integration of a practical skill set. Considering the recent creation of the BSPH degree, students graduating with the BSPH will most benefit from preparation for successful transition into the workforce or further academic study. Successes from this learning modality should be applied to other undergraduate degree programs to ensure readiness for entrance into the academic arena or public health workforce.

## Author Contributions

BS is the director of the capstone BSPH evidence-based course. BS has seen many iterations of this course and is able to offer suggestions and expertise as far as how it should be taught. VF and CM both taught this class for 3 years while going through their Doctor of Public Health degree programs and are able to offer insight into how a practice-based degree feeds into the teaching of an undergraduate practice-based course.

## Conflict of Interest Statement

The authors declare that the research was conducted in the absence of any commercial or financial relationships that could be construed as a potential conflict of interest.
